# Stabilization of Symptomatic Bone Marrow Metastases in Breast Cancer: A Case Report of Two Patients Treated With Trastuzumab Deruxtecan

**DOI:** 10.7759/cureus.87118

**Published:** 2025-07-01

**Authors:** Nektarios Alevizopoulos, Dimitrios Alexandris, Vaios Oreopoulos, Michail G Pavlakis, Georgios Kanellopoulos

**Affiliations:** 1 Oncology, Evangelismos General Hospital, athens, GRC; 2 Internal Medicine, Evangelismos General Hospital, Athens, GRC; 3 Oncology, Evangelismos General Hospital, Athens, GRC; 4 Oncology, BIOCLINIC General Hospital of Athens, Athens, GRC

**Keywords:** bone marrow infiltration, breast cancer outcomes, enhertu (fam-trastuzumab deruxtecan), her2-negative breast cancer, trastuzumab-deruxtecan

## Abstract

Symptomatic bone marrow infiltration (BMI) in breast cancer is exceedingly rare and often associated with poor prognosis due to cytopenias and limited therapeutic options. We report two elderly patients with a history of hormone receptor-positive, HER2-negative breast cancer who presented with pancytopenia years after initial treatment. Bone marrow biopsy revealed HER2-low metastatic relapse. Treatment with trastuzumab deruxtecan (T-DXd) led to unexpectedly rapid and complete hematologic recovery after only two cycles, with both patients remaining stable and free of marrow disease for over two years. These cases demonstrate the remarkable efficacy and tolerability of T-DXd in this uncommon and challenging clinical scenario, highlighting its potential as a valuable therapeutic option in HER2-low breast cancer with BMI.

## Introduction

Breast cancer (BC) ranks as the second most prevalent malignancy worldwide, with projections indicating approximately 2,296,840 new cases and 666,103 fatalities anticipated in 2024 [1]. The classification of BC encompasses various categories based on the expression of several receptors, including estrogen receptors (ER), progesterone receptors (PR), and the human epidermal growth factor receptor 2 (HER2) [2]. It is estimated that 15-20% of all BC cases are characterized by the overexpression or amplification of the HER2 gene [3].

The determination of HER2 status is facilitated through immunohistochemistry (IHC) and in situ hybridization (ISH). A positive HER2 status is denoted as IHC 3 (+) or 2 (+)/ISH positive, while a negative HER2 status is classified as IHC (0). A newly recognized subgroup, termed HER2-low, encompasses neoplasms exhibiting lower detectable levels of HER2, specifically IHC 2 (+)/ISH negative or IHC 1 (+) [4]. HER2-low tumors, previously classified as HER2 negative, represent a substantial segment of the BC population, estimated at approximately 50%, and may derive benefit from HER2-targeted therapies [4, 5].

BC is prone to relapse, with 70% of recurrences manifesting as new metastatic sites [6, 7]. Although bone marrow (BM) metastases are relatively common, symptomatic bone marrow infiltration (BMI) is exceedingly rare, occurring in less than 0.2% of all cases involving BM metastases [8, 9].

BMI is diagnosed through a BM biopsy, a procedure of critical importance, particularly when the patient presents with pancytopenia [10]. There exists a paucity of data regarding the management of symptomatic BMI in BC patients, especially given that most anti-cancer pharmacotherapies may exacerbate the total blood count [10].

Targeted therapies against HER2-expressing cancer cells have profoundly transformed the landscape of BC, leading to substantial reductions in recurrence rates and significantly enhancing patient survival [11]. Trastuzumab deruxtecan (T-DXd) represents a novel class of antibody-drug conjugate, comprising a humanized anti-HER2 monoclonal antibody linked to a topoisomerase I inhibitor cytotoxic payload [11]. The primary objective of T-DXd is to selectively deliver antitumoral agents to cells that express the HER2 receptor, thereby integrating antibody-dependent cellular cytotoxicity with traditional chemotherapeutic agents [11].

Although T-DXd has been extensively utilized in patients with HER2-positive BC, it only recently garnered approval for specific patients with HER2-low metastatic BC (August 2022 in the United States, January 2023 in Europe). The phase 2 DAISY study and the phase 3 DESTINY-Breast04 study underscore the efficacy of T-DXd in the management of metastatic and/or unresectable HER2-low BC patients [12, 13].

To date, there has been a dearth of data concerning BC with symptomatic BMI, particularly regarding therapeutic management. In this report, we elucidate two rare cases of metastatic BC patients exhibiting pancytopenia who were successfully treated with T-DXd.

## Case presentation

We hereby present two female patients with a history of lobular BC who underwent total mastectomy and were subsequently monitored for their disease. Both individuals, aged over 72, had received adjuvant chemotherapy and were currently undergoing treatment with the aromatase inhibitor letrozole. Their surgical specimens revealed resected ER-positive, PR-negative, and HER2-negative (IHC 0) malignancies. Throughout an extensive monitoring period of 8 and 9 years, neither patient exhibited any imaging or laboratory findings indicative of metastatic BC.

**Figure 1:**
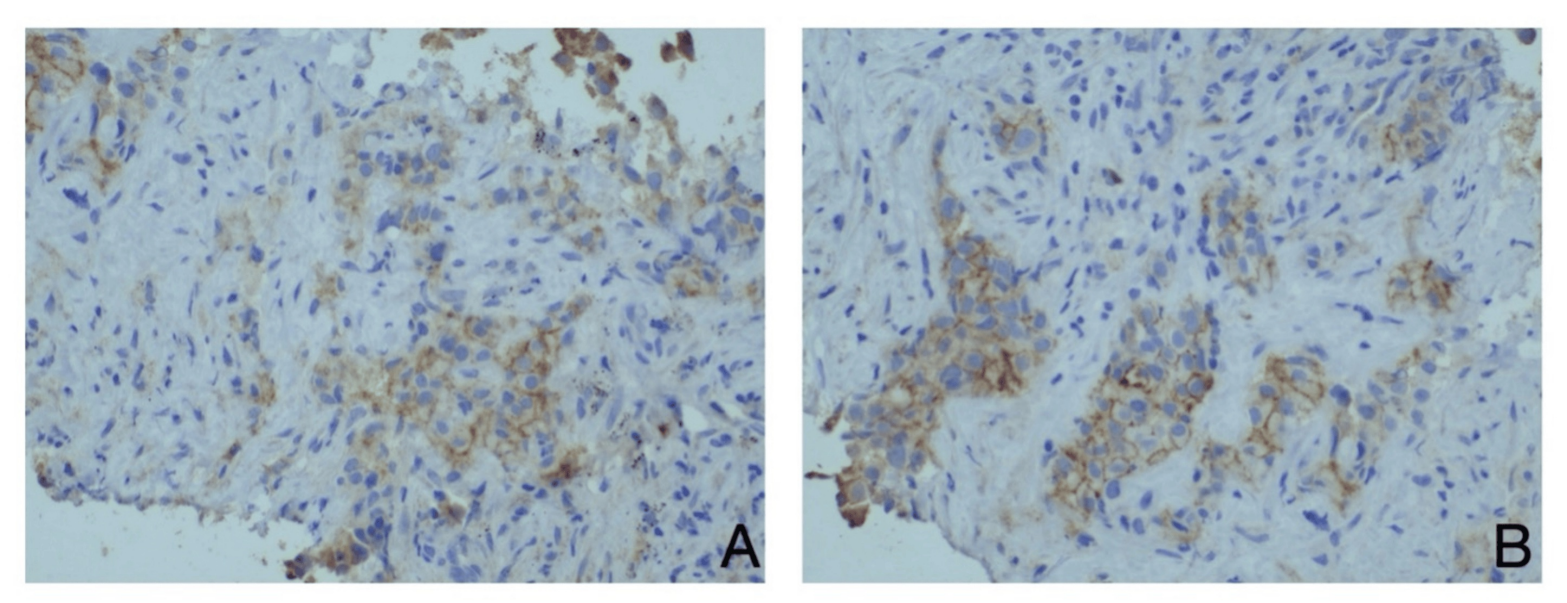
The bone marrow biopsy disclosed the presence of metastatic breast cancer cells. A comprehensive pathological analysis, incorporating immunohistochemistry, was conducted, revealing HER2/neu (IHC 1+) characterized by incomplete membrane staining that is subtle and scarcely perceptible in over 10% of tumor cells (ASCO/CAP HER2 Testing Guideline Update / 2022).

However, both patients presented with thrombocytopenia, characterized by platelet counts below 60,000, in conjunction with mild anemia. They were evaluated for the potential of a secondary hematological malignancy; however, BM aspiration revealed infiltration by BC with low HER2 staining (Figure 1). In light of the absence of established chemotherapy protocols or alternative therapies for their revised HER2 status, we elected to administer T-DXd (5.6 mg/kg every 21 days intravenously).

It was unexpectedly remarkable to observe a mild yet complete recovery of both platelet and hemoglobin levels following merely two subsequent infusions of T-DXd. As of today, two years post-initiation of therapy, the patients remain free of BM disease and continue their treatment regimen.

## Discussion

Metastatic involvement of the skeletal system is a prevalent occurrence in BC, with approximately 65-75% of all patients with metastatic BC exhibiting bone metastases. However, in numerous instances, these metastases remain asymptomatic. In contrast, complete BMI is a rare yet grave phenomenon, resulting in clinically significant impairments in hematopoiesis. This condition may manifest as anemia, thrombocytopenia, leukopenia, or more intricate disorders such as leukoerythroblastosis, and in severe cases, even pancytopenia [14]. This caution is particularly salient in the context of patients who already exhibit compromised hematopoiesis. Numerous authors have underscored the potential application of various therapeutic modalities, including low-dose capecitabine, endocrine therapy, trastuzumab therapy, or cyclin-dependent kinase 4-6 inhibitors [9, 10, 15-18].

In conjunction with the administration of anti-cancer therapies, the incorporation of bisphosphonates is advocated as an integral component of the management strategy for bone metastasis [17]. Although the efficacy of bisphosphonates in preventing osseous metastases in high-risk early-stage BC remains ambiguous, research conducted by Solomayer et al. has demonstrated that treatment with zoledronic acid can facilitate the eradication of disseminated tumor cells within the BM of patients suffering from metastatic BC [19]. This finding intimates a potential role for bisphosphonates not merely in managing the complications associated with bone metastases but also in targeting residual malignancy within the BM, thereby underscoring the necessity for further exploration into their broader implications in BC therapeutics.

Our patients exhibited advanced metastatic BC characterized by symptomatic BM involvement and a low-HER2 profile, despite their protodiagnostic histological records indicating a HER2-negative status (IHC 0). Consequently, we instituted T-DXd as an integral component of the second-line therapeutic strategy. Initially, there were reservations regarding the treatment due to the potential adverse effects associated with T-DXd, which include neutropenia (16%), anemia (7%), and leukopenia (6%) [20]. However, the overall efficacy of T-DXd facilitated a gradual improvement in the patients’ hematological profiles, alleviating initial concerns and highlighting the potential advantages of this therapy in similar clinical scenarios.

There has been considerable interest in identifying factors that influence the overall survival of patients with BC and BM involvement. Research indicates that several pivotal factors play a role, including performance status, platelet count, and erythroblast count in peripheral blood at the diagnosis of BM involvement [10]. Among the hematologic cell lines, red blood cells are frequently the first to be impacted by BM involvement, culminating in anemia [10]. This early effect on red blood cells is a hallmark of BM suppression induced by metastatic infiltration, and effectively managing the hematologic complications is paramount for optimizing patient outcomes.

## Conclusions

These cases underscore the importance of carefully balancing therapeutic risks and benefits in patients with advanced disease and complex presentations such as symptomatic BMI. They highlight the value of a multidisciplinary, patient-centered approach and the need for ongoing surveillance to elucidate the long-term safety and efficacy of novel targeted therapies. The incorporation of emerging biomarkers and advanced imaging techniques may further refine patient selection and optimize treatment strategies, reducing toxicity while enhancing clinical outcomes. Upholding ethical principles and ensuring informed consent remain fundamental to engaging patients meaningfully in their care.
